# Diorganotin(IV) Derivatives of *N*-Methyl *p*-Fluorobenzo-Hydroxamic Acid: Preparation, Spectral Characterization, X-ray Diffraction Studies and Antitumor Activity

**DOI:** 10.3390/molecules18078696

**Published:** 2013-07-22

**Authors:** Naqeebullah, Yang Farina, Kok Meng Chan, Lo Kong Mun, Nor Fadilah Rajab, Theng Choon Ooi

**Affiliations:** 1 School of Chemical Sciences and Food Technology, Faculty of Science & Technology, Universiti Kebangsaan Malaysia, 43600 Bangi, Selangor Darul Ehsan, Malaysia; 2 Department of Chemistry, Faculty of Basic Sciences, University of Balochistan, 87300 Sariab Road Quetta, Pakistan; 3 Environmental Health Programme, Faculty of Allied Health Sciences, Universiti Kebangsaan Malaysia, Jalan Raja Muda Abdul Aziz, 50300 Kuala Lumpur, Malaysia; 4 Department of Chemistry, Faculty of Science, University of Malaya, 50603 Kuala Lumpur, Malaysia; 5 Biomedical Science Programme, Faculty of Health Sciences, Universiti Kebangsaan Malaysia, Jalan Raja Muda Abdul Aziz, 50300 Kuala Lumpur, Malaysia

**Keywords:** organotin, hydroxamic acid, X-ray diffraction, antitumor activity

## Abstract

Three diorganotin(IV) complexes of the general formula *R*_2_Sn[*R*cC(O)N(*R*N)O] (*R*c = aryl, *R*N = Alkyl) have been synthesized by refluxing in toluene the corresponding diorganotin(IV) oxides with the free ligand *N*-methyl *p*-fluorobenzohydroxamic acid, using a Dean and Stark water separator. The ligand was derived from the reaction of the corresponding *p*-fluorobenzoyl chloride and *N*-methylhydroxylamine hydrochloride in the presence of sodium hydrogen carbonate. The isolated free ligand and its respective diorganotin compounds have been characterized by elemental analysis, IR and ^1^H-, ^13^C-, ^119^Sn-NMR spectroscopies. The crystal structures of the diorganotin complexes have been confirmed by single crystal X-ray diffraction methods. The investigations carried out on the diorganotin(IV) complexes of *N*-methyl *p*-fluorobenzohydroxamic acid confirmed a 1:2 stoichiometry. The complex formation took place through the *O*,*O*-coordination via the carbonyl oxygen and subsequent deprotonated hydroxyl group to the tin atom. The crystal structures of three diorganotin complexes were determined and were found to adopt six coordination geometries at the tin centre with coordination to two ligand moieties.

## 1. Introduction

Hydroxamic acids [1], *R*cC(O)N(*R*N)OH (*R*c = alkyl/aryl; *R*N = alkyl/aryl or H), have been the source of much biochemical interest in recent years reflecting the fact that they demonstrate a wide variety of biological activities. Much of their activities are due to their chelating properties with metal ions, especially with transition metals, hence constituting a very important class of chelating agents with versatile biological activities [2,3]. The principal coordination mode observed in metal-hydroxamic acid complexes is the *O*,*O*-bidentate chelation in which the ligand is either singly deprotonated (hydroxamato) or doubly (*R*N = H) deprotonated (hydroximato) [4]. A number of synthetic routes are available for the preparation of hydroxamic acids [5,6,7,8,9,10], but some of them are tedious, time consuming and also costly. The reasonable way of producing hydroxamic acid derivative is the reaction of hydroxylamine with acid chlorides or esters [11]. Hydroxamic acids are capable of the inhibition of a variety of enzymes, including ureases [12,13], peroxidases [14], and matrix metalloproteinases (MMP) [15,16] and are also capable of competing as siderophores for iron-(III) [17,18]. These compounds are used in the design of therapeutics targeting cancer [19,20], cardiovascular diseases [21], HIV [22], Alzheimer’s [23], malaria [24,25], and allergic diseases [26]. 

Organotin(IV) complexes with bidentate O-donor ligands [27], including *N*-substituted hydroxamic acids, are well known and have been a continuing subject of study in the recent years [28], highlighting the synthesis of a number of complexes with interesting properties [29,30,31]. Moreover, some of the diorganotin(IV) hydroxamates have been structurally characterized by X-ray diffraction studies, which are well documented in the literature [32,33,34]. Organotin compounds are widely studied class of organometallic compounds with, broad spectrum of applications, being used in antifouling paints [35], as homogeneous catalysts [36] and in agriculture that give rise to ubiquitous environmental contamination [37,38]. The biological activity of organotin compounds is predominantly determined by the number and nature of organic groups linked to the central tin atom and generally decreases in the following order: R_3_Sn^+^ > R_2_Sn^2+^ > RSn^3+^ [29,39,40,41]. In addition, the increasing interest in the chemistry of organotin(IV) compounds has led to the extended studies against cancer [42,43].

The structural and biological diversity of organotin hydroxamates stirred our interest to further illustrate the coordination chemistry and anti-proliferative activity of organotin compounds with hydroxamic acid, herein we report the synthesis of a new ligand *N*-methyl *p-*fluorobenzohydroxamic acid (L_H_) and its diorganotin(IV) derivatives (CH_3_)_2_SnL_2_, (C_4_H_9_)_2_SnL_2_ and (C_6_H_5_)_2_SnL_2_ with interesting structural features to expand their scope.

## 2. Results and Discussion

### 2.1. Synthesis

**Scheme 1:**
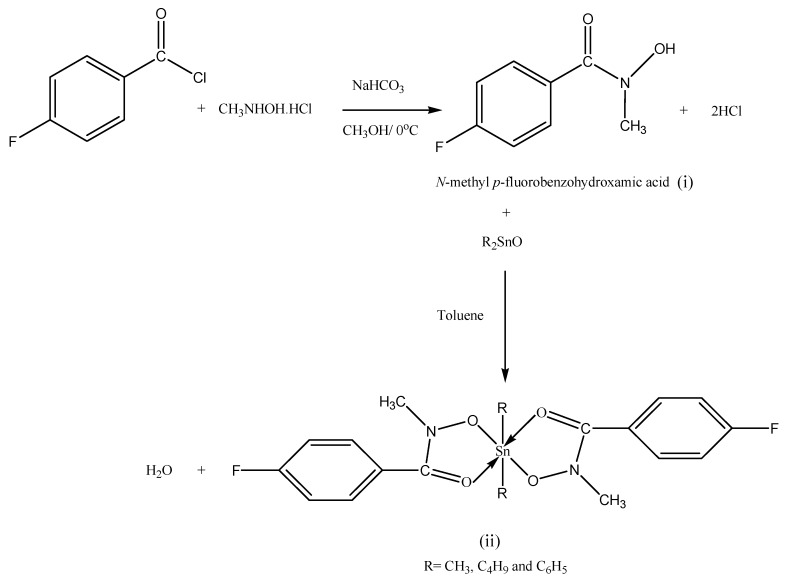
A general reaction scheme of the free ligand (**i**) and its diorganotin(IV) complexes (**ii**).

The ligand was prepared by the reaction of *p*-fluorobenzoyl chloride with *N*-methyl-hydroxylamine hydrochloride in the presence of sodium hydrogen carbonate as catalyst. All the reagents were in the same ratio by weight (i). Diorganotin(IV) complexes were synthesized in 2:1 molar ratio, by refluxing the free ligand with diorganotin(IV) oxides in hot toluene for 5–6 h with stirring and the water formed was removed azeotropically using a Dean-Stark apparatus (ii), as summarized in Scheme 1. The resulting solution was cooled and filtered and the solvent evaporated. The solid was precipitated by adding petroleum ether (60–80 °C) and then recrystallized from ethanol. The purity of the ligand and the diorganotin complexes were assured by TLC analysis using silica gel-G as adsorbent.

The newly synthesized ligand and its diorganotin complexes are white or colorless solids, stable in air and soluble in common organic solvents. Tin was determined gravimetrically, by igniting a known quantity of each complex. The calculated values were in a good agreement with the experimental values. 

### 2.2. Infra-Red Spectroscopy

Solid state infrared spectra of the *N*-methyl *p*-fluorobenzohydroxamic acid and its complexes have been recorded in the range 4,000–400 cm^−1^. The principal infrared absorption bands are those due to ν(O-H), ν(C=O), ν(C-N) and ν(N-O) stretching vibrations of the hydroxamate group observed in the spectrum of free hydroxamic acid at 3,175, 1,610, 1,432 and 908 respectively. The ν(O-H) band is observed in the range 3,175 cm^−1^ appeared as a broad band indicating the presence of extensive hydrogen bonding. The characteristic band ν(C=O) positioned within the range 1,610 cm^−1^ is notably, below the usual ketonic ν(C=O) range of 1,650 cm^−1^ [44,45], indicates that in the solid state the ligand exist in the keto form.

The IR spectra of the free ligand and its diorganotin(IV) complexes illustrated clear differences. In all cases, (O-H) stretching modes were absent in the spectra of the complexes, thus suggesting the deprotonation of the hydroxamate group on complexation, Similarly, the (C=O) group are shifted to lower frequencies in the range 1,599–1,602 cm^−1^, indicating a further shift of (C=O) to lower energy thus suggesting the predominance of the enolic form to give a five membered chelate rings at the tin centre. Moreover, the (N-O) stretching vibrations occurring at 938–948 cm^−1^ in the diorganotin(IV) hydroxamates, are shifted to higher frequencies, excluding the coordination via the nitrogen atom [46]. The occurrence of (Sn-O) in the range of 474–453 cm^−1^ indicates the chelation of the tin centre to the enolate oxygen [45,47].

### 2.3. NMR Spectroscopy

^1^H-NMR spectra for the investigated ligand and their organotin(IV) complexes have been recorded in CDCl_3_ solution and tetramethylsilane as internal standard at room temperature. In the ^1^H-NMR spectra the free ligand show a signal at 10.34 ppm, which is due to the intramolecularly hydrogen bonded hydroxyl proton. The peak disappeared in the ^1^H-NMR spectra of the complexes indicating, thereby, the substitution of the hydroxyl proton and chelation of the oxygen to the tin atom. The proton signals appearing in the region 3.40 ppm were attributed to methyl protons attached to the nitrogen atom, which remained unchanged on chelation, supporting further, the non-involvement of this group in complexation. In the dimethyltin(IV) derivative, the proton resonances appeared as a singlet in the region 0.713 ppm, with well-defined satellites. The value of the two bond coupling constant ^2^*J*(^119^Sn-^1^H) calculated from tin satellites in the ^1^H-NMR spectra of dimethyltin(IV) complex was found in the region of 84.22 Hz, and the estimated C-Sn-C bond angle is 136.4°, based on the equation of Lockhart and Manders [Equation (1)] [48], which fall in the region for six-coordinate tin [49]. In the dibutyltin(IV) complex, the butyl protons were found as a multiplet and a triplet in the regions 1.36–1.84 ppm and 0.88 ppm due to -(CH_2_)_3_ and the terminal CH_3_ respectively. A complex multiplet found at 8.17–8.32 ppm for the aromatic protons of the free ligand and all complexes, is due to the overlapping of the signals of the aromatic protons of the ligand and phenyl group protons in diphenyltin(IV) complex [50,51].

*θ*(C-Sn-C) = 0.0161(^2^*J*_Sn-H_)^2^ – 1.32 (^2^*J*_Sn-H_) + 133.4 (1)

^13^C-NMR spectra for the investigated ligand and its organotin(IV) complexes have been recorded in CDCl_3_ solution and tetramethylsilane as internal standard at room temperature. ^13^C-NMR chemical shifts in every complex showed the expected resonances with appropriate multiplicities and intensities and the spectra are generally in agreement with the results drawn from ^1^H-NMR signals. The carbonyl (C=O) signal appeared at 165.0 ppm in free ligand and were shifted upfield in the corresponding complexes (164.9–161.3ppm), indicating a decrease in electron density at the carbon atom when oxygen atom is chelated to the tin atom. The methyl carbon attached to the nitrogen appears at 38.37–40.98 ppm. In dimethyltin (IV) complex, the methyl carbon attached to the tin appeared at 6.57 ppm and the observed ^119^Sn satellites in ^13^C-NMR spectrum provide ^1^*J*(^13^C-^119^Sn) coupling constant value 785.27 Hz, and the estimated C-Sn-C bond angle is 145.7°, based on the equation of Lockhart and Manders [Equation (2)] [48], which is of the same order of magnitude of those observed in hexa-coordinate organotin(IV) compounds. The butyl carbons attached to the tin in dibutyltin(IV) complex appeared at 13.85, 26.63, 26.85 and 27.44 ppm. The signals appeared at 115–163 ppm, were assigned to the aromatic carbons. By comparing the ^13^C-NMR spectra of the free ligand with its diorganotin (IV) complexes, a slight upfield shift has been observed in the position of carbonyl signal, suggesting the bidentate nature of hydroxamate group. One can notice that the oxygen chelated to metal ion reduce the electron density at carbon atom, hence considered the cause for chemical shift [52,53].

(^1^*J*_Sn-C_) = 11.4 *θ* − 875 (2)

The ^119^Sn-NMR spectra of diorganotin(IV) complexes studied herein in DMSO, at room temperature. The ^119^Sn-NMR chemical shifts of organotin(IV) compounds appear to depend not only on the coordination number, on the other hand also on the alkyl groups bound to the metal ion and the types of donor atoms [54]. The spectra show one sharp signal in dimethyl-, dibutyl- and diphenyltin complexes at 𝛿 = −407 ppm, −367 ppm and −205 ppm respectively, which strongly supports the six coordination around tin in a distorted octahedral geometry [55,56,57]. In the later an associated structure such as the stereoisomers specie is thus present in solution similar to spectra have reported by [56,58].

### 2.4. X-ray Crystallography

**Figure 1:**
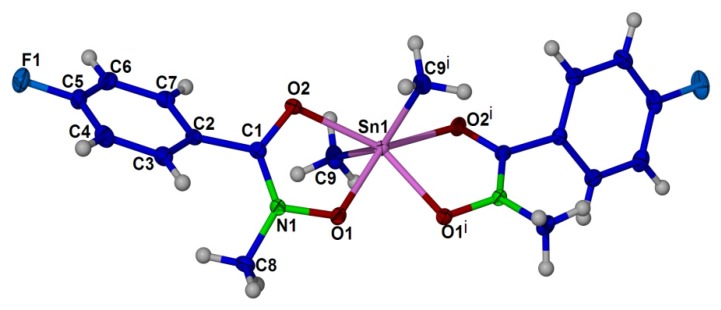
Thermal ellipsoidal plot of C_18_ H_20_ F_2_ N_2_ O_4_ Sn (compound **1**). Displacement ellipsoids are drawn at the 50% probability level, and H atoms are shown as spheres of arbitrary radii. Symmetry transformation code i: −x + 1,y,−z + 3/2.

**Figure 2:**
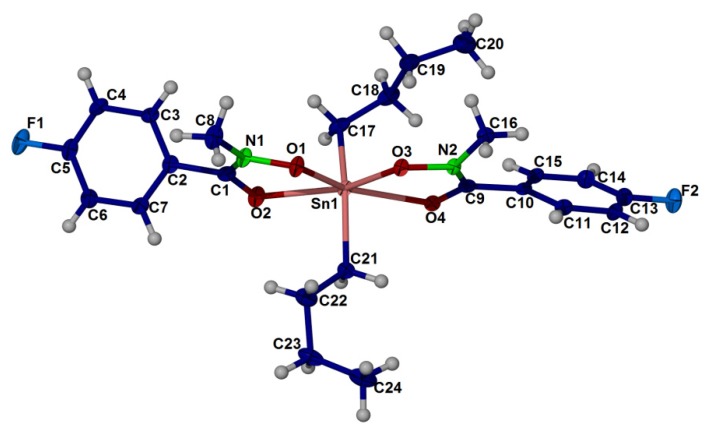
Thermal ellipsoidal plot of C_24_ H_32_ F_2_ N_2_ O_4_ Sn (compound **2**). Displacement ellipsoids are drawn at the 50% probability level, and H atoms are shown as spheres of arbitrary radii.

**Figure 3:**
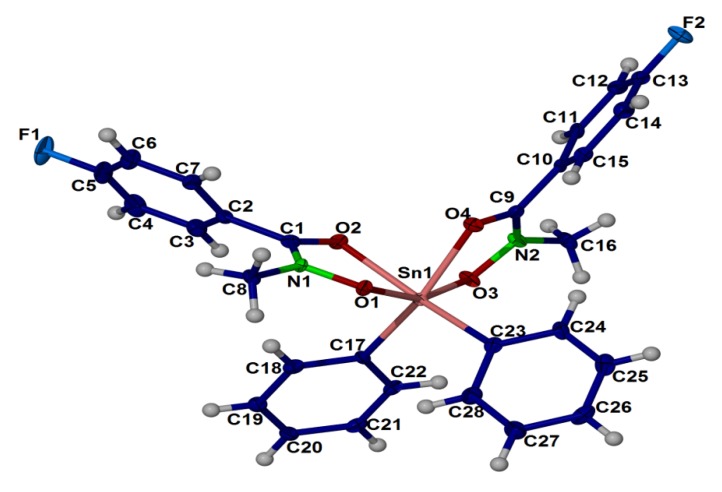
Thermal ellipsoidal plot of C_28_ H_24_ F_2_ N_2_ O_4_ Sn (compound **3**). Displacement ellipsoids are drawn at the 50% probability level, and H atoms are shown as spheres of arbitrary radii.

The crystal structure of compound (**1**), (**2**) and (**3**) are shown in Figure 1, Figure 2, Figure 3, respectively. Selected bond angles and bond lengths are presented in Table 1, Table 2. The molecular structures of these diorganotin complexes showed that the tin atom is bonded to two *N*-methyl-*p*-fluorobenzohydroxamic acids via the hydroxyl oxygen and the carbonyl oxygen [30,59]. The two organic groups of the diorganotin fragment complete the six coordination geometry at tin for the three complexes. It is evident that the carbonyl oxygen are weakly coordinated to the tin compared to the covalent Sn-O_hydroxyl_ bonds [compound (**1**): Sn-O1 2.0921(9), 2.0921(9) and Sn-O2 2.3778(9), 2.3778(9); compound (**2**): Sn-O1: 2.117(3), 2.132(3) and Sn-O2 2.356(3), 2.407(3); compound (**3**): Sn-O1 2.110(2), 2.103(2) and Sn-O2 2.221(2), 2.183(2)]. The bond distances and angles of the three complexes as given in Table 1, Table 2 revealed that the geometry of the crystals is distorted octahedral around the six coordinated tin(IV) ion, similar to the diphenyltin(IV) bis(*N*-methyl *p*-bromobenzohydroxamate) [27] and di-*n*-butyl-(4-chlorobenzo-hydroxamato)tin(IV) [60]. The distortion in the coordination sphere of the metal ion from the ideal geometry may be due to the structural constraints imposed by the hydroxamic acid ligand framework. The ligand bite angles O1-Sn-O2 at tin for the three complexes are small with the values of 71.22(3)°, 71.23(3)° for (1), 70.36(9)°, 71.96(10)° for (2) and 73.26(8)°, 74.15(8)° for (3). Interestingly, the two alkyl substituents of the diorganotin fragment in compounds (**1**) and (**2**) adopt the *trans* conformation [C-Sn-C angle of (1) and (2) is 143.98(8)° and 141.2(2)°, respectively] whereas the two phenyl substituent in complex (**3**) adopts the *cis* conformation [C-Sn-C angle is 104.2(1)°].

**Table 1:** Selected bond lengths (Å) of the complexes (**1**), (**2**) and (**3**).

(1)		(2)		(3)	
Sn1-O1 2.0921(9)	F1-C5 1.3583(15)	Sn1 O1 2.117(3)	N1 C1 1.312(5)	Sn1 O3 2.103(2)	N1 C1 1.326(4)
Sn1-O1 i 2.0921(9)	O1-N1 1.3807(13)	Sn1 C21 2.125(4)	N1 O1 1.385(4)	Sn1 O1 2.110(2)	N1 O1 1.379(3)
Sn1-C9 i 2.1184(13)	O2-C1 1.2696(15)	Sn1 O3 2.132(3)	N1 C8 1.453(5)	Sn1 C17 2.144(3)	N1 C8 1.452(4)
Sn1-C9 2.1184(13)	N1-C1 1.3192(16)	Sn1 C17 2.143(4)	N2 C9 1.321(5)	Sn1 C23 2.156(3)	N2 C9 1.322(4)
Sn1-O2 2.3778(9)	N1-C8 1.4558(16)	Sn1 O2 2.356(3)	N2 O3 1.375(4)	Sn1 O4 2.183(2)	N2 O3 1.380(3)
Sn1-O2 i 2.3778(9)		Sn1 O4 2.407(3)	O2 C1 1.262(5)	Sn1 O2 2.221(2)	O2 C1 1.277(4)
		F1 C5 1.354(5)	O4 C9 1.263(5)	F1 C5 1.353(4)	O4 C9 1.285(4)

**Table 2:** Selected bond angles (deg) of the complexes (**1**), (**2**) and (**3**).

(1)		(2)		(3)		
O1-Sn1-O1i 76.58(5)	C9i-Sn1-O2i 82.95(4)	O1 Sn1 C21 105.01(13)	C9 N2 O3 119.0(3)		O3 Sn1 O1 158.08(9)	C9 N2 O3 117.6(2)
O1-Sn1-C9i 97.86(5)	C9-Sn1-O2i 85.64(4)	O1 Sn1 O3 76.51(10)	O3 N2 C16 111.9(3)		O3 Sn1 C17 89.96(10)	O1 N1 C8 112.8(2)
O1i-Sn1-C9i 110.40(5)	O2-Sn1-O2i 142.49(4)	C21 Sn1 O3 103.19(13)	N1 O1 Sn1 114.8(2)		O1 Sn1 C17 103.49(10)	O3 N2 C16 112.7(2)
O1-Sn1-C9 110.40(5)	N1-O1-Sn1 113.09(7)	O1 Sn1 C17 104.85(14)	C1 O2 Sn1 110.1(2)		O3 Sn1 C23 105.08(10)	C1 O2 Sn1 112.7(18)
O1i-Sn1-C9 97.86(5)	C1-O2-Sn1 107.76(8)	C21 Sn1 C17 141.14(16)	C9 O4 Sn1 111.7(2)		O1 Sn1 C23 88.55(10)	N1 O1 Sn1 113.1(16)
C9i-Sn1-C9 143.98(8)	C1-N1-O1 118.20(10)	O3 Sn1 C17 107.52(14)	N2 O3 Sn1 117.7(2)		C17 Sn1C23 104.16(11)	N2 O3 Sn1 113.1(16)
O1-Sn1-O2 71.22(3)	C1-N1-C8 128.68(11)	O1 Sn1 O2 71.96(10)	C18 C17 Sn1 118.9(3)		O3 Sn1 O4 74.15(8)	C9 O4 Sn1 113.8(18)
O1i-Sn1-O2 145.75(3)	O1-N1-C8 112.43(10)	C21 Sn1 O2 85.83(13)	Sn1 C17 H17A 107.6		O1 Sn1 O4 88.51(8)	C22 C17 Sn1 120.5(2)
C9i-Sn1-O2 85.64(4)	O2-C1-N1 120.10(12)	O3 Sn1 O2 148.46(10)	Sn1 C17 H17B 107.6		C17 Sn1 O4 159.85(10)	C18 C17 Sn1 122.0(2)
C9-Sn1-O2 82.95(4)	O2-C1-C2 119.21(11)	C17 Sn1 O2 80.36(14)	C22 C21Sn1 117.2(3)		C23 Sn1 O4 92.10(10)	C24 C23 Sn1 126.3(2)
O1-Sn1-O2i 145.75(3)	N1-C1-C2 120.64(11)	O1 Sn1 O4 146.74(9)	Sn1 C21 H21A 108.0		O3 Sn1 O2 90.35(8)	C28 C23 Sn1 116.7(2)
O1i-Sn1-O2i 71.23(3)	F1-C5-C6 118.22(13)	C21 Sn1 O4 80.23(12)	Sn1 C21 H21B 108.0		O1 Sn1 O2 73.26(8)	O2 C1 N1 118.9(3)
		O3 Sn1 O4 70.36(9)	O2 C1 N1 121.1(4)		C17 Sn1 O2 87.69(10)	F1 C5 C6 118.8(3)
		C17 Sn1 O4 88.16(13)	F1 C5 C6 118.9(4)		C23 Sn1 O2 160.33(10)	F1 C5 C4 118.1(3)
		O2 Sn1 O4 141.15(9)	F1 C5 C4 117.7(4)		O4 Sn1 O2 80.26(8)	O4 C9 N2 118.8(3)
		C1 N1 O1 118.8(3)	O4 C9 N2 120.1(3)		C1 N1 O1 117.6(2)	
		O1 N1 C8 111.7(3)	O4 C9 C10 119.2(3)			

Symmetry transformation code for compound (**1**) i: −x + 1, y, −z + 3/2.

### 2.5. Antitumor Activity *in vitro*

The synthesized organotins were evaluated for the biological activity, specifically cytotoxicity on HCT116 colorectal carcinoma cell line. All the tested organotins induced a concentration-dependent anti-proliferative effect towards HCT116 cells upon treatment for 24 h. However, the cytotoxicity of dibutyltin(IV)Bis[*N*-methyl *p*-fluorobenzohydroxamate] could not be assessed due to the lack of solubility in DMSO at room temperature. Triphenyltin(IV) *N*-methyl *p*-fluorobenzohydroxamate was the most potent organotin with IC_50_ value of 0.41 µM, follow by diphenyltin(IV)bis[*N*-methyl *p*-fluorobenzohydroxamate] and dimethyltin(IV)bis[*N*-methyl *p*-fluorobenzohydroxamate] as shown in Table 3. Our current data are in agreement with previous study, whereby the triphenyltin(IV) complexes exhibit higher antiproliferative effects compare to diphenyltin(IV) complexes [61,62,63]. Similarily, it has also been demonstrated that triphenyltin(IV) complex possess the highest cytotoxic effect whereas the dimethyltin(IV) complex have little or no cytotoxic effect on HCT116 cells upto 250 µM treatment for 24 h [64]. Therefore, triphenyltin(IV) *N*-methyl *p*-fluorobenzohydroxamate has the potential to be developed as an anti-tumor agent due to the potent cytotoxic effect at nano molar concentration which warrant further mechanistic studies.

**Table 3:** IC_50_ values of organotins on HCT116 cells.

Compounds	IC_50_ values (µM)
dimethyltin(IV)bis[*N*-methyl *p*-fluorobenzohydroxamate]	>40
diphenyltin(IV)bis[*N*-methyl *p*-fluorobenzohydroxamate]	2.45
dibutyltin(IV)bis[*N*-methyl *p*-fluorobenzohydroxamate]	NA
triphenyltin(IV)*N*-methyl *p*-fluorobenzohydroxamate	0.41

## 3. Experimental

### 3.1. General

The chemicals were purchased from Aldrich and were used as received. All the chemicals were of analytical grade. The triphenyltin(IV) *N*-methyl *p*-fluorobenzohydroxamate was success-fully prepared according to a standard method reported in the literature [65]. The melting points were determined in open capillary tubes using an Electrothermal 9300 digital melting point apparatus. The percentage compositions of the elements (CHN) for the compounds were determined using an elemental analyzer CHNS-O Model Fison EA 1108. Solid state infrared spectra of the compounds are recorded in the range 4000–400 cm^−1^. The infrared spectra were recorded as potassium bromide discs using a Perkin-Elmer spectrophotometer GX. The ^1^H-, ^13^C- and ^119^ Sn-nuclear magnetic resonance spectra were recorded using the Bruker FT-NMR 600 MHz Cryo-Prob spectrometer and the JEOL JNM-ECP 400 spectrometer using DMSO/CDCl_3_ as a solvent and tetramethylsilane as an internal standard. Crystals structures determination were carried out on a Bruker Smart APEX CCD area detector diffractometer equipped with graphite mono-chromatised Mo-Kα (λ = 0.71073Å) radiation in each case. All data collection was carried out at 100K. The program *APEX2* (Bruker [66]) was used for collecting frames of data, indexing of reflections and determination of lattice parameters, *SAINT* (Bruker 2008) for absorption correction, and SHELX97 (Sheldrick [67]). HCT116 human colorectal carcinoma cells were obtained from the American Type Culture Collection (Manassas, Virginia, USA). The cells were grown in McCoy’s 5A medium (Invitrogen Cooperation, Paisley, UK) supplemented with 10% FBS (PAA Laboratories, Morningside, QLD, Australia) and maintained at 37 °C with 5% CO_2_ in humidified incubator.

### 3.2. Synthesis of Ligand

*p*-Fluorobenzoyl chloride (0.01 mol) was poured down drop by drop to a stirred cold solution of *N*-methylhydroxylamine hydrochloride (0.01 mol) containing sodium hydrogen carbonate (0.01 mol) and was further stirred for 30 min below 4 °C. The solution was filtered and reduced to evaporate at low pressure. The precipitate was then dissolved in boiling ethyl acetate to remove any undissolved substance and then the filtrate is placed in the fridge overnight to obtain the crystals.

*N-methyl p-fluorobenzohydroxamic acid*. (**HL**): Colourless crystals. Yield: 84%. Melting point: 88–89 °C. ^1^H-NMR [DMSO-d_6_]: 𝛿 (ppm) = 10.34 (s, br, 1H, O-H), 7.11–7.57 (m, 4H, C_6_H_4_), 3.40 (s, 3H, N-CH_3_). ^13^C-NMR [DMSO-d_6_]: 𝛿 (ppm) = 165.0 (CO); 163–115 (C aromatic); 38.37 (C aliphatic). IR (KBr pellets, cm^−1^): 3175(s, br, ν O-H), 1610 (s, ν C=O), 1432 (s, ν C-N) and 908 (s, ν N-O). Elemental Analysis: Calcd. (%) for H_8_C_8_NO_2_F (molecular weight: 169.06): C, 56.80; H, 8.28; N, 4.73. Found (%): C, 57.08; H, 8.36; N, 5.14.

### 3.3. Synthesis of Complexes

Diorganotin(IV) complexes were synthesized by 2:1 molar ratio, dissolving the free ligand (0.005 mol) in hot toluene and then added the diorganotin(IV) oxides (0.0025 mol) to the solution. The solution was refluxed for 5–6 h with magnetic stirrer and the water formed during the course of reaction was removed azeotropically using a Dean-Stark apparatus. The solution was then cooled and filtered. The filtrate was placed under vacuum to evaporate the solvent and the solid was precipitated by adding petroleum ether (60–80 °C) and then recrystallized in ethanol.

*Dimethyltin(IV)Bis[N-methyl p-fluorobenzohydroxamate]* (**1**). Colourless crystals. Yield: 71%. Melting point: 115–116 °C. ^1^H-NMR [DMSO-d_6_]: 𝛿 (ppm) = 7.11–7.77 (m, 4H, C_6_H_4_), 3.41 (s, 3H, N-CH_3_), 0.71 (s, 3H, Sn-CH_3_), ^13^C-NMR [DMSO-d_6_]: 𝛿 (ppm) = 161.3 (CO), 124–131 (C aromatic), 39.29 (N-C), 6.57 (Sn-C). ^119^ Sn-NMR [DMSO-d_6_]: 𝛿 (ppm) = −407. IR (KBr pellets, cm^−1^): 1600 (s, ν C=O), 1432 (s, ν C-N), 938 (s, ν N-O), 439 (s, ν Sn-O) and 576 (s, ν Sn-C). Elemental Analysis: Calcd. (%) for H_20_C_18_N_2_O_4_F_2_Sn (molecular weight: 485.16): C, 44.53; H, 4.13; N, 5.77; Sn, 24.53. Found (%): C, 43.74; H, 5.78; N, 5.00; Sn, 22.05.

*Dibutyltin(IV)Bis[N-methyl p-fluorobenzohydroxamate]* (**2**). Colourless crystals. Yield: 77%. Melting point: 103–104°C. ^1^H-NMR [DMSO-d_6_]: 𝛿 (ppm) = 7.11–7.44 (m, 4H, C_6_H_4_), 3.45 (s, 3H, N-CH_3_), 1.36–1.84 (m, 6H, Sn-CH_2_-CH_2_-CH_2_), 0.88 (t, 3H, -CH_3_). ^13^C-NMR [DMSO-d_6_]: 𝛿 (ppm) = 164.5 (CO), 115–129 (C aromatic), 40.98 (N-C), 13.85–27.44 (Sn-C). ^119^Sn-NMR [DMSO-d_6_]: 𝛿 (ppm) = −367. IR (KBr pellets, cm^−1^): 1600 (s, ν C=O), 1530 (s, ν C-N), 953 (s, ν N-O), 474 (s, ν Sn-O) and 562 (s, ν Sn-C). Elemental Analysis: Calcd. (%) for H_32_C_24_N_2_O_4_F_2_ Sn (molecular weight: 569.26): C, 50.59; H, 5.85; N, 4.92; Sn, 20.90. Found (%): C, 50.16; H, 4.91; N, 5.85; Sn, 19.21.

*Diphenyltin(IV)Bis[N-methyl p-fluorobenzohydroxamate]* **(3)**. White crystals. Yield: 82%. Melting point: 203–204 °C. ^1^H-NMR [DMSO-d_6_]: 𝛿 (ppm) = 8.17–8.32 (m, 9H, C_6_H_4_, C_6_H_5_), 3.46 (s, 3H, N-CH_3_). ^13^C-NMR [DMSO-d_6_]: 𝛿 (ppm) = 164.9 (C=O), 123–150 (C aromatic), 38.86 (N-C). ^119^ Sn-NMR [DMSO-d_6_]: 𝛿 (ppm) = −205. IR (KBr pellets, cm^−1^): 1599 (s, ν C=O), 1454 (s, ν C-N), 948 (s, ν N-O), 453 (s, ν Sn-O) and 563 (s, ν Sn-C). Elemental Analysis: Calcd. (%) for H_24_C_28_N_2_O_4_F_2_Sn (molecular weight: 609.19): C, 55.16; H, 3.97; N, 4.60; Sn, 19.54. Found (%): C, 55.64; H, 4.74; N, 4.02; Sn, 18.60.

### 3.4. X-ray Crystallography

The single crystals of dimethyltin, dibutyltin and diphenyltin complexes of *N*-methyl *p*-fluorobenzohydroxamic acid of suitable quality were each mounted on a fine glass capillary and aligned on the Bruker SMART APEX2 diffractometer, equipped with graphite monochromated Mo-*Kα* radiation source (λ = 0.71073 Å). The range of theta for data collections together with other crystallographic information are given in Table 4. All calculations were performed using the SHELXTL-97 package [68]. Crystallographic data for the compounds (**1**), (**2**) and (**3**) have been deposited with the Cambridge Crystallographic Data Centre, CCDC reference numbers (924068, 933217, 924061). This information may be obtained free of charge from: the Director, CCDC, 12 Union Road, Cambridge, CB2 1EZ, UK (fax: +44-1223-336033; e-mail:deposit@ccdc.cam.ac.uk; website: http://www.ccdc.cam.ac.uk).

**Table 4:** Crystallographic parameter for the diorganotin compounds (**1**), (**2**) and (**3**).

Compound	(1)	(2)	(3)
Gross formula	C_18_ H_20_ F_2_ N_2_ O_4_ Sn	C_24_ H_32_F_2_ N_2_ O_4_ Sn	C_28_ H_24_ F_2_ N_2_ O_4_ Sn
*M*	485.05	569.21	609.15
Crystal system, space group	Monoclinic, *C2/c*	Triclinic, *P-1*	Triclinic, *P-1*
Crystal shape	Block	Block	Block
Colour	Colourless	Colourless	White
*a*, Ǻ	21.7581(2)	11.0271(7)	8.8999(2)
*b*, Ǻ	11.2694(1)	11.1155(7)	12.3601(3)
*c,* Ǻ	7.8964(1)	11.1404(7)	12.4362(3)
*α*, deg	90	75.948(3)	109.600(1)
*β*, deg	94.357	82.636(3)	99.770(1)
*γ*, deg	90	77.919(3)	98.015(1)
*V,* Ǻ ^3^	1930.58(3)	1290.99(14)	1241.34(5)
*Z*	4	2	2
*d_c_,* g/cm^-3^	1.669	1.464	1.37
F(000)	968	580	612
Μ, mm^-1^	1.368	1.035	1.083
*T* , K	100(2)	100(2)	100(2)
Crystal size, mm	0.24× 0.29× 0.35	0.40× 0.15× 0.05	0.10×0.05× 0.05
*T*_min_	0.6415	0.6824	0.8995
*T*_max_	0.7457	0.9501	0.9479
measured reflections	8979	10517	10184
independent reflections	2221	5056	4581
reflections with I > 2s(I)	2191	4593	4231
*R*_int_	0.0114	0.1236	0.0219
*θ*_max_	27.5	26	25.5
*θ*_min_	1.88	1.89	1.79
Completeness to theta	0.998	0.998	0.993
*h*	−28 28	−12 13	−10 10
*k*	−14 14	−13 13	−14 14
*l*	−10 10	−13 13	−15 15
*R*[*F*^2^ > 2s(*F*^2^)]	0.0136	0.0539	0.0239
*wR*(*F*^2^)	0.0367	0.1444	0.0654
*S*	1.146	1.096	1.19
reflections	2221	5056	4581
parameters	125	302	336
restraints	0	0	0
ρ_max_ e Ǻ^−3^	0.261	1.538	0.532
∆ρ_min_ e Ǻ^−3^	−0.450	−3.311	−0.571

*w* = 1/[σ^2^ (*F*_o_^2^) + (0.0717*P*)^2^ + 0.215*P*] where *P* = (*F*_o_^2^ + 2*F*_c_^2^)/3 for compound (**1**); *w* = 1/[σ^2^(*F*_o_^2^) + (0.0921*P*)^2^ + 0.0000*P*] where *P* = (*F*_o_^2^ + 2*F*_c_^2^)/3 for compound (**2**); *w* = 1/[σ^2^(*F*_o_^2^) + (0.0454*P*)^2^ + 0.5345*P*] where *P* = (*F*_o_^2^ + 2*F*_c_^2^)/3 for compound (**3**).

### 3.5. MTT Cytotoxicity Assay

The antitumor activity against carcinoma cells was assayed by the MTT method [69]. Cells were seeded in 96-well plate at a density of 5 × 10^4^ cells per well in a volume of 200 mL and were treated with various concentrations of the compounds for 24 h. After treatment, 20 µL of 5 mg/mL MTT (Sigma-Alrich, St. Louis, MO, US) was added to each treated cells and further incubated for 4 h at 37 °C. Subsequently the medium was discarded from each well before adding 200 µL DMSO (Fisher Scientific, Loughborough, UK). For complete dissolution, the plate was incubated for 15 min followed with gentle shaking for 5 min. The cytotoxic effect of the organotins on HCT116 cells was assessed by measuring the absorbance of each well at 570 nm. Mean absorbance for each concentration was expressed as a percentage of vehicle control absorbance and plotted versus compound concentration.

## 4. Conclusions

In this work, we have successfully synthesized a novel ligand and its three diorganotin(IV) hydroxamates, which gave fairly sharp melting points indicating that the compounds were pure and were characterized by elemental analyses, IR, NMR and X-ray single-crystal diffraction. The structural analyses of complexes **1**–**3** reveal that the coordination mode observed in metal-hydroxamic acid complexes is the *O*,*O*-bidentate chelation and a five membered chelated ring was assembled. The NMR and X-ray studies were in full concurrent with the IR spectral evidences. The crystal structures of the three diorganotin complexes adopted a six coordination geometry at tin which is coordinated to the carbonyl oxygen and hydroxyl oxygen of two benzohydroxamic acid ligands and the two organic substituent of the diorganotin(IV) fragment. The diphenyltin(IV) and triphenyltin(IV) complexes demonstrated promising antiproliferative activities whereas dimethyltin(IV) shows very little cytotoxic effect at μM concentration on human HCT116 cells. 

## Supplementary Materials

Supplementary materials can be accessed at: http://www.mdpi.com/1420-3049/18/7/8696/s1.
