# Supplementary figures and images for: Diorganotin(IV) Derivatives of N-Methyl p-Fluorobenzo-Hydroxamic Acid: Preparation, Spectral Characterization, X-ray Diffraction Studies and Antitumor Activity
Source: Molecules. 2013 Jul 22;18(7):8696–711. doi: 10.3390/molecules18078696 (PMC6270222; doi:10.3390/molecules18078696)

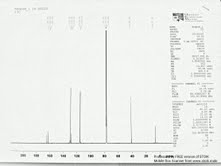

Supplement: Supplementary file 1 [file molecules-18-08696-s001.zip › Manuscript Supplementary Files/download (1).jpg]

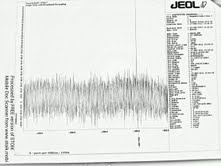

Supplement: Supplementary file 1 [file molecules-18-08696-s001.zip › Manuscript Supplementary Files/download.jpg]

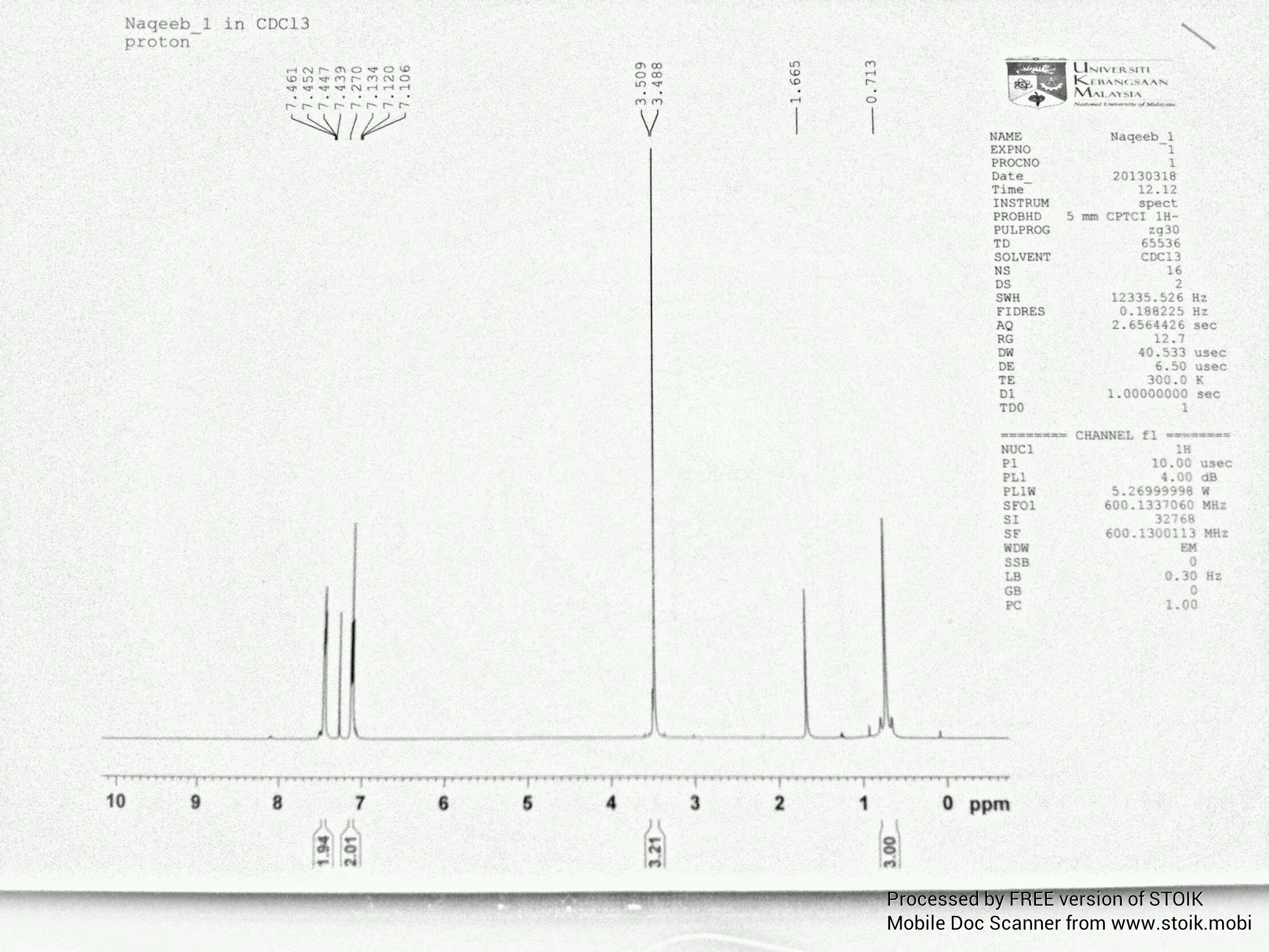

Supplement: Supplementary file 1 [file molecules-18-08696-s001.zip › Manuscript Supplementary Files/page_12.jpg]

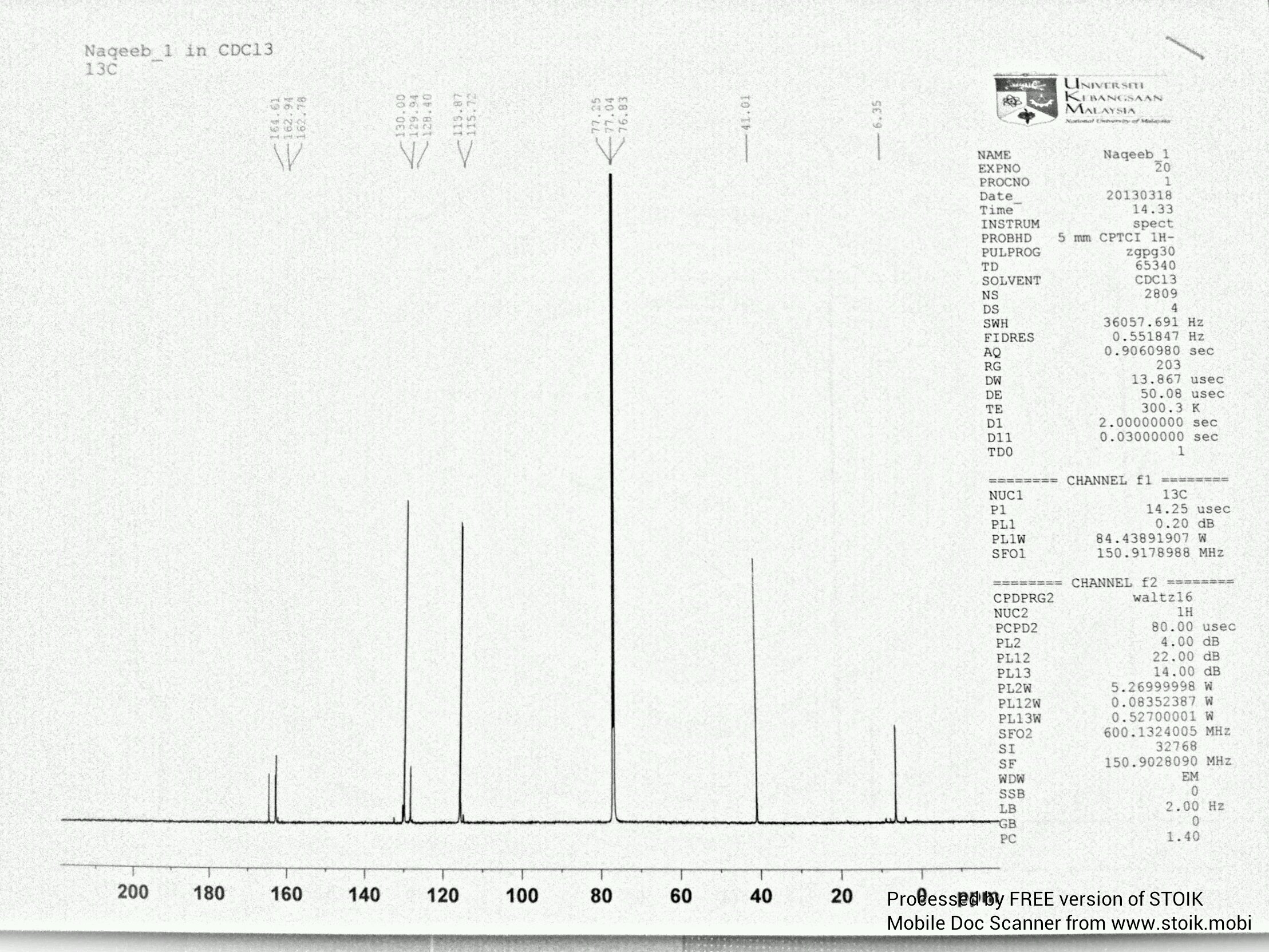

Supplement: Supplementary file 1 [file molecules-18-08696-s001.zip › Manuscript Supplementary Files/page_13.jpg]

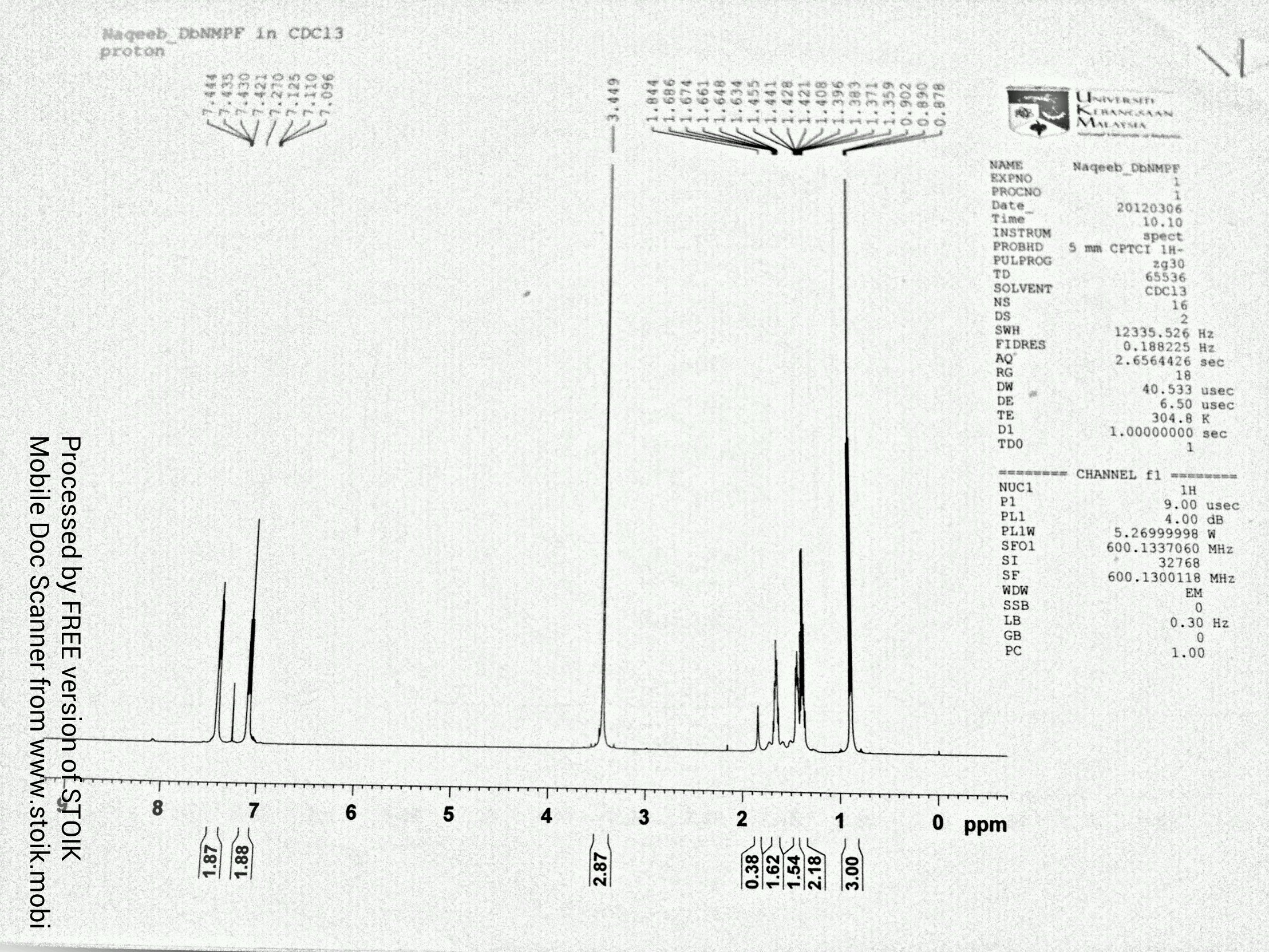

Supplement: Supplementary file 1 [file molecules-18-08696-s001.zip › Manuscript Supplementary Files/page_5.jpg]

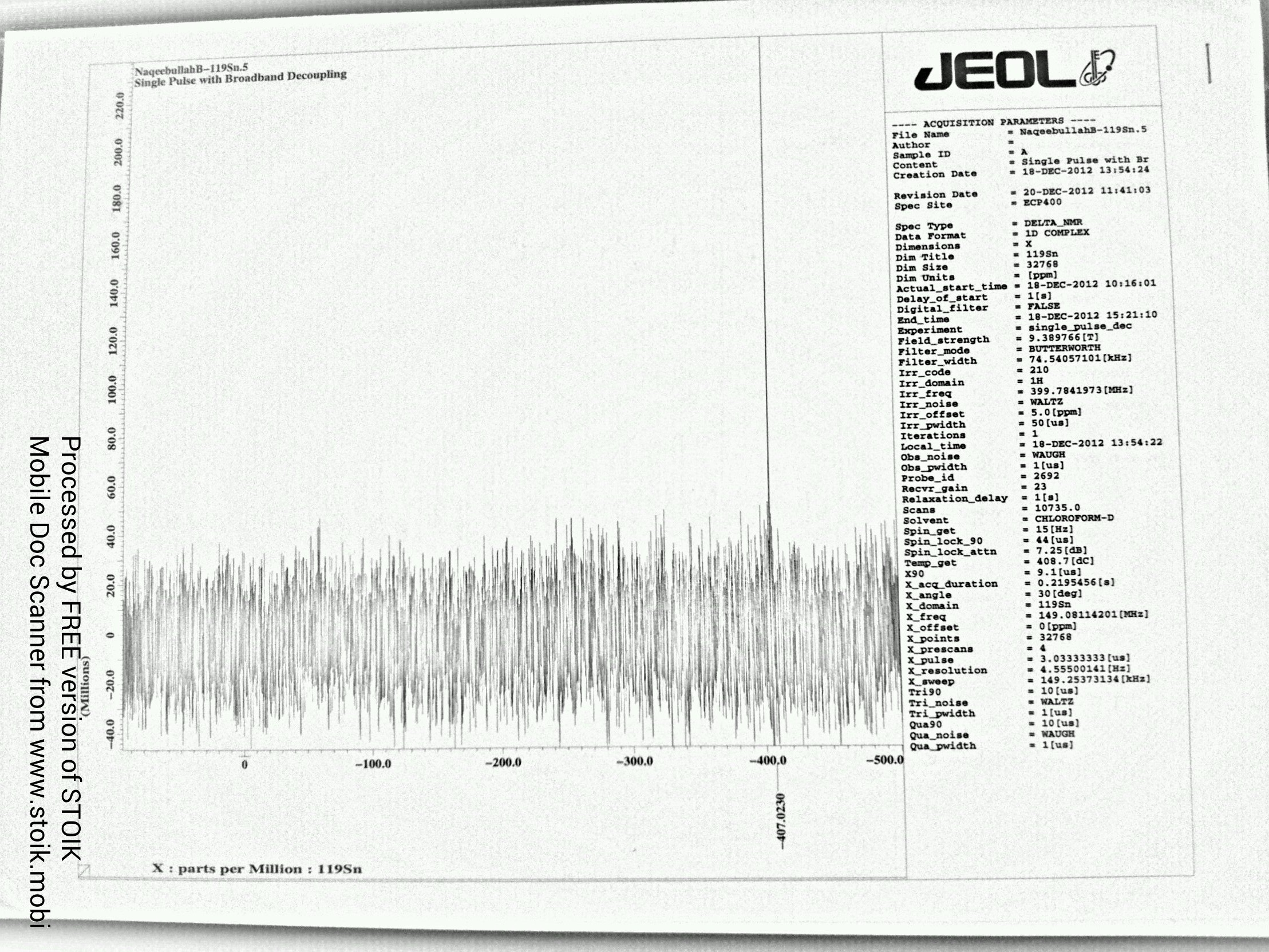

Supplement: Supplementary file 1 [file molecules-18-08696-s001.zip › Manuscript Supplementary Files/page_9.jpg]
